# Early tumor shrinkage as a predictor of favorable outcomes in patients with advanced pancreatic cancer treated with FOLFIRINOX

**DOI:** 10.18632/oncotarget.12007

**Published:** 2016-09-13

**Authors:** Yasuhiro Kaga, Yu Sunakawa, Yutaro Kubota, Teppei Tagawa, Taikan Yamamoto, Toshikazu Ikusue, Yu Uto, Kouichirou Miyashita, Hirokazu Toshima, Kouji Kobayashi, Atsushi Hisamatsu, Wataru Ichikawa, Takashi Sekikawa, Ken Shimada, Yasutsuna Sasaki

**Affiliations:** ^1^ Divison of Medical Oncology, Showa University Northern Yokohama Hospital, Yokohama, Japan; ^2^ Division of Medical Oncology, Showa University School of Medicine, Tokyo, Japan; ^3^ Divison of Medical Oncology, Showa University Koto Toyosu Hospital, Tokyo, Japan; ^4^ Division of Medical Oncology, Showa University Fujigaoka Hospital, Yokohama, Japan

**Keywords:** pancreatic cancer, FOLFIRINOX, early tumor shrinkage

## Abstract

There are several reports on the correlation between early tumor shrinkage (ETS) or depth of response (DpR) and survival in chemotherapies for colorectal cancer; however, few studies have investigated it in pancreatic cancer. We therefore investigated whether the ETS will predict outcomes in 59 patients with advanced pancreatic cancer treated with FOLFIRINOX therapy. The association of ETS with progression-free survival (PFS) and overall survival (OS) was evaluated but also we addressed to the correlation between outcomes and DpR. ETS was defined as a reduction ≥ 20% of target lesions' diameters measured at 6 to 8 weeks from treatment start. DpR was percentage of maximal tumor shrinkage observed at the nadir diameter compared with baseline. Among 47 evaluable patients for the ETS, 12 (25.5%) patients experienced ETS. The ETS was significantly associated with better PFS (9.0 vs. 4.2 months) as well as OS (24.0 vs. 9.1 months); moreover, the association had a statistically significance for PFS but a strong trend for OS in multivariate analysis. The DpR was statistically significantly but weakly associated with OS. In conclusion, this is the first report that the early response to chemotherapy may predict favorable outcomes in patients with advanced pancreatic cancer treated with FOLFIRINOX therapy.

## INTRODUCTION

Pancreatic cancer is the fourth leading cause of death from cancer over the world, which is usually a high grade of malignancy at the diagnosis and is one of intractable cancers [1]. It carries a severe prognosis, which 5-year survival rate was reported to be lower than 5% [1]. In addition, the morbidity of the disease is increasing in recent year; therefore, more than 30,000 people die of pancreatic cancer every year in Japan [2]. Advanced pancreatic cancer remains an incurable disease with few cure treatments. The phase III PRODIGE 4/ACCORD 11 trial provided one of current standard regimens for advanced pancreatic cancer, consisting of oxaliplatin, irinotecan, fluorouracil, and leucovorin (FOLFIRINOX), which has superior response rate (RR) and survival benefit even with severe toxicity in patients with advanced pancreatic cancer. The RR, median progression-free survival (PFS), and overall survival (OS) were significantly better in patients receiving FOLFIRINOX therapy compared to patients receiving gemcitabine therapy (RR, 31.6% vs. 9.4%; median PFS, 6.4 months vs. 3.3 months, HR 0.47, 95% CI 0.37–0.59, *P* < 0.001; median OS, 11.1 months vs. 6.8 months, HR 0.57, 95% CI 0.45–0.73, *P* < 0.001) [3]. Since then, FOLFIRINOX regimen has been used as a standard first-line treatment for patients with advanced pancreatic cancer; however, there are still no biomarkers to predict favorable outcomes of the FOLFIRINOX. There have been several reports on the correlation between early tumor shrinkage (ETS) or depth of response (DpR) and survival time in chemotherapies for metastatic colorectal cancer [4–7]. The phase III TRIBE trial compared first-line FOLFOXIRI plus bevacizumab with FOLFIRI plus bevacizumab for unresectable metastatic colorectal cancer patients. In a subgroup analysis of the TRIBE trial, a highly significant correlation of both ETS and DpR with survival parameters (PFS, post progression survival, and OS) was reported in the arm of triplet plus bevacizumab treatment [5]. Moreover, a subgroup analysis of the phase III FIRE-3 trial which evaluated first-line FOLFIRI plus cetuximab versus FOLFIRI plus bevacizumab also demonstrated a strong correlation of ETS with PFS and OS [8]. Recently, Heinemann V et al. reviewed the ETS and DpR for colorectal cancer treatment and evaluated their potential as predictive markers for the clinical management. The review showed that the ETS not only relates to a prognostic category but also distinguishes patients with high sensitivity to treatment and more favorable prognosis from a heterogeneous group of patients classified as no ETS [4]. An observational cohort study for advanced pancreatic cancer patients treated with the triplet regimen has revealed Eastern Cooperative Oncology Group (ECOG) performance status (PS), liver metastases, and neutrophil-to-lymphocyte ratio as the most important predictors of survival [9]. However, to the best of our knowledge, there have been few studies to investigate the association between ETS or DpR and clinical outcomes of chemotherapy in pancreatic cancer. We therefore investigated whether the ETS will predict outcomes in patients with advanced pancreatic cancer treated with FOLFIRINOX therapy.

## RESULTS

### Patient characteristics

Between November 2012 and November 2015, 59 patients were enrolled from 3 institutions of Showa University (Showa University Northern Yokohama Hospital, Showa University Hospital, and Showa University Koto Toyosu Hospital). The patient characteristics at baseline are shown in Table 1. The median age was 63 years (range, 34–76), forty-four patients (74.6%) had a PS of 0. The primary site of tumor was the head of pancreas in 26 patients (44.1%). Thirteen patients (22.0%) had a biliary stent, and 47 patients (79.7%) had metastatic diseases. Forty patients (67.8%) were treated with FOLFIRINOX as a first-line treatment. In the all population, RR, median PFS, and OS were 27.3%, 5.3 months (95% CI, 4.2–7.9), and 10.3 months (95% CI, 7.6–11.1), respectively.

**Table 1:** Demographic and baseline characteristics of patients (*n* = 59)

Characteristics	
Age-year	
Median	63
Range	34–76
Sex-no.(%)	
Male	40 (67.8)
Female	19 (32.2)
ECOG PS-no.(%)	
0	44 (74.6)
1	15 (25.4)
Diagnosis-no (%)	
Locally advanced	12 (20.3)
Metastatic	47 (79.7)
Synchronous	38
Metachronous	9
Treatment line-no (%)	
First-line	40 (67.8)
Second-line	19 (32.2)
Pancreatic tumor location-no.(%)	
Head	26 (44.1)
Body	22 (37.3)
Tail	9 (15.3)
Multicentric	2 (3.4)
Biliary Stent-no.(%)	
Yes	13 (22.0)
No	46 (78.0)

### Association between response to the treatment and clinical outcomes

Among all enrolled patients, 4 patients were not evaluable for response due to patients with locally advanced diseases or peritoneal diseases. Among 55 evaluable patients for response, 15 (27.3%) patients achieved a partial response. The OS was statistically significantly longer in responders compared to non-responders (24.0 months vs. 9.0 months, HR 0.33, 95% CI 0.12–0.75, log-rank *P* = 0.010). No significant association was observed between response and PFS (Table 2).

**Table 2:** Associations of ETS and response with clinical outcomes

	ETS+ (*n* = 12)	ETS- (*n* = 35)	HR (95% CI)	*P*-value
PFS	9.0 months	4.2 months	0.43 (0.18-0.91)	0.032
OS	24.0 months	9.1 months	0.36 (0.12–0.87)	0.031

	Responder (*n* = 15)	Non-responder (*n*= 40)	HR (95% CI)	*P*-value
PFS	5.7 months	4.2 months	0.56 (0.27–1.07)	0.090
OS	24.0 months	9.0 months	0.33 (0.12–0.75)	0.010

ETS, early tumor shrinkage; PFS, progression-free survival; OS, overall survival.

**Figure 1:**
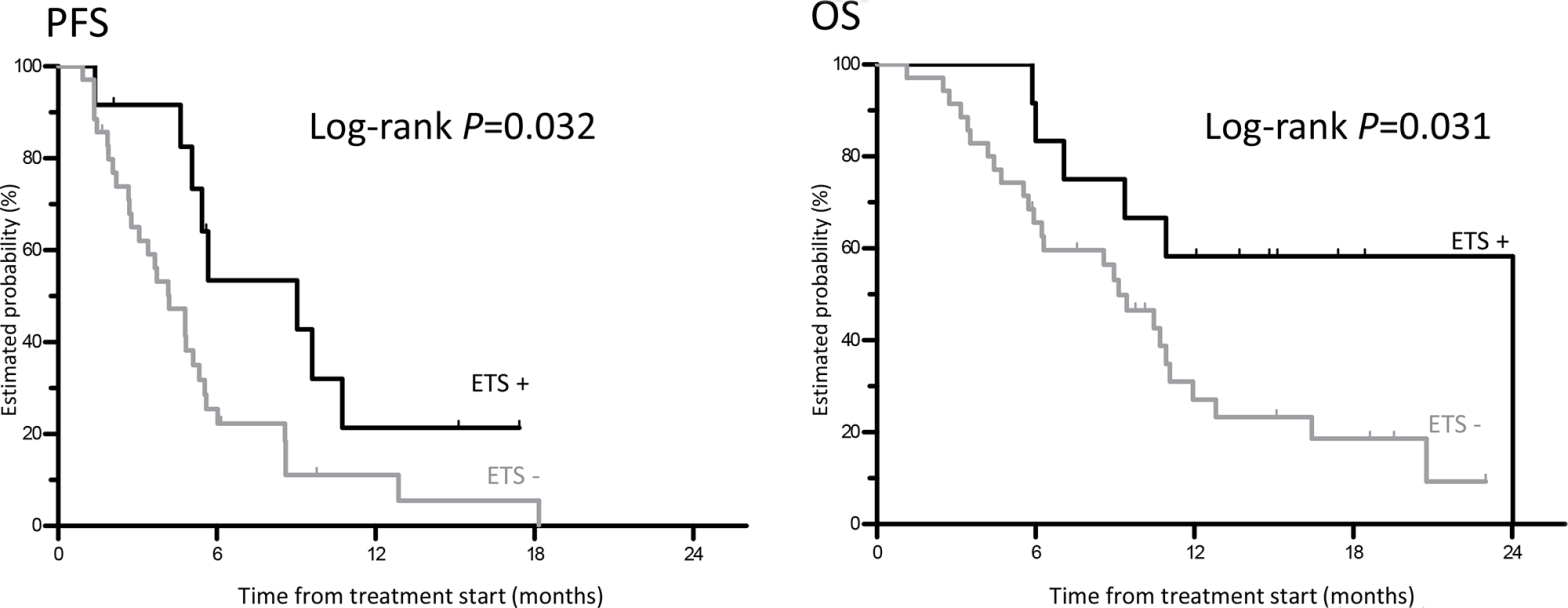
Kaplan-Meier curves for progression-free survival and overall survival in relation to early tumor shrinkage

Forty-seven (79.6%) of all 59 patients had measurable tumors for the ETS. Among the evaluable patients for the ETS, 12 (25.5%) patients experienced an ETS. The PFS was statistically significantly longer in patients with ETS compared to those with no ETS (9.0 months vs. 4.2 months, HR 0.43, 95% CI 0.18–0.91, log-rank *P* = 0.032). Moreover, patients with ETS had a significantly better OS (24.0 months vs 9.1 months, HR 0.36, 95% CI 0.12–0.87, log-rank *P* = 0.031) (Table 2, Figure 1).

### Association between DpR and clinical outcomes

**Figure 2:**
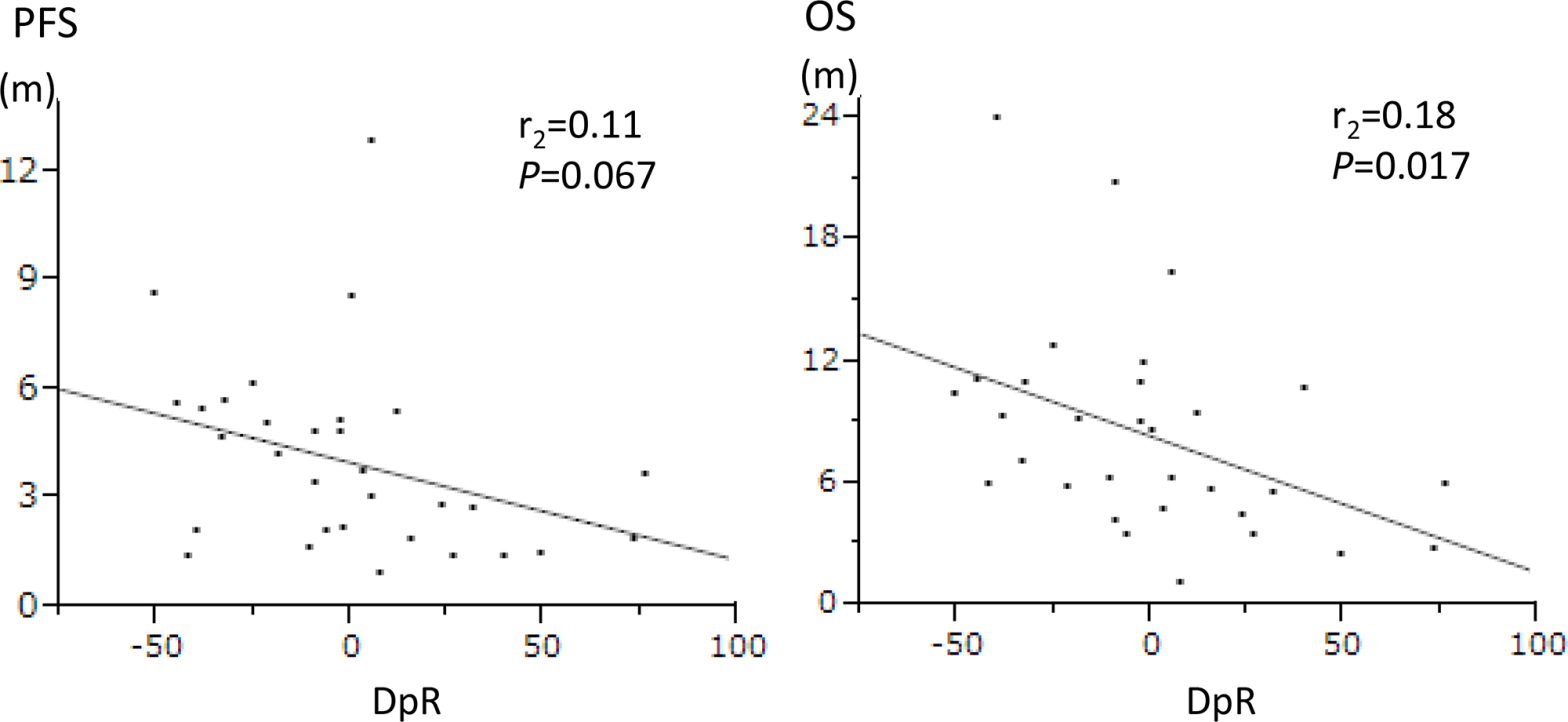
The correlation of depth of response (DpR) with clinical outcomes by the Spearman's rank correlation coefficient

Thirty-one patients (52.5%) who experienced an OS event were estimated for the DpR. Median DpR was −10.3% (from −75.7 to 100). The DpR was statistically significantly but weakly associated with OS (*r*_s_ = 0.18, *P* = 0.017) and was not associated with PFS (Figure 2).

### Multivariate analysis

We performed a multivariate analysis for several variables including response and ETS to precisely evaluate the association with clinical outcomes. In the multivariate analysis adjusted for ECOG PS and diagnosis, the response was statistically significantly associated with both PFS and OS (HR 0.38; 95% CI 0.18–0.80; *P* = 0.011, HR 0.33; 95% CI 0.13–0.79; *P* = 0.013, respectively). On the contrary, the ETS had a significant association with PFS (*P* = 0.02) but a strong trend with OS (*P* = 0.065) (Table 3).

**Table 3:** Multivariate analysis

Variables	Progression-free survival				Overall survival	
	HR	95% CI	*P*-value*	HR	95% CI	*P*-value*
Gender						
Male vs. Female	1.06	0.56–2.00	0.87	0.93	0.48–1.81	0.82
ECOG PS						
0 vs. 1	0.56	0.29–1.09	0.086	0.45	0.22–0.91	0.026
Diagnosis						
Locally advanced vs. metastatic	0.32	0.12–0.84	0.021	0.33	0.11–0.94	0.037
Response						
Responder vs. non-responder	0.38	0.18–0.80	0.011	0.33	0.13–0.79	0.013
ETS						
ETS+ vs. ETS–	0.37	0.16–0.85	0.020	0.40	0.15–1.06	0.065

* *P* values were based on Wald test for PFS and OS in the multivariable Cox regression model adjusted for ECOG performance status (0 vs. 1) and diagnosis (locally advanced vs. metastatic).

## DISCUSSION

We analyzed the associations between ETS or DpR and clinical outcomes of FOLFIRINOX treatment in patients with advanced pancreatic cancer. To the best of our knowledge, the present analysis is the first attempt to evaluate the association of response to the intensive chemotherapy with survival parameters in pancreatic cancer. Our data showed that response had a significant correlation with OS in the assessable population. Moreover, the ETS was significantly associated with both PFS and OS, suggesting that achieving rapid and deeper response may lead not only to delay of disease progression but also to prolonged survival.

FOLFIRINOX is now considered to be one of first-line standard treatments for patients with advanced pancreatic cancer according to results of the phase III trial by Conroy et al. [3]. However, it cannot be applied to all patients with pancreatic cancer due to severe toxicity of the FOLFIRINOX, such as neutropenia, leucopenia, febrile neutropenia, anorexia, and diarrhea. The study by Metges et al. who are the original investigators in the PRODIGE 4/ACCORD 11 trial, based on their established criteria, showed that 81% of 242 patients required a dose reduction (median dose intensity; 5-FU bolus 82%, oxaliplatin 78%, irinotecan 81%), but that this result did not affect treatment efficacy compared to the PRODIGE 4/ACCORD 11 trial: RR 39% vs. 32%, median PFS 6.5 vs. 6.4 months, and median OS 10.9 vs. 11.1 months [10]. A Japanese phase II trial of the FOLFIRINOX showed that grade 3 or 4 neutropenia and febrile neutropenia were more frequently observed compared to those in the global phase III trial (77.8% vs. 45.7%, 22.2% vs. 5.4%, respectively) [11]. Recently, several clinical studies of a modified FOLFIRINOX regimen have been carried out to reduce its toxicities. Blazer et al. reported their experience with reducing irinotecan in addition to dropping 5-FU bolus, and also concluded that the modified regimen was effective and well tolerated with no episodes of grade 3 or 4 neutropenia and thrombocytopenia, but with 46% of patients requiring a dose reduction for other toxicities [12]. Another Japanese phase II trial which evaluated the efficacy and safety of modified FOLFIRINOX regimen with intravenous oxaliplatin 85 mg/m^2^, irinotecan 150 mg/m^2^, 5-FU infusion 2400 mg/m^2^ over 46 hours, and no bolus 5-FU, demonstrated that the modified regimen has an improved safety profile with maintained efficacy in advanced pancreatic cancer [13]. We therefore clinically utilized irinotecan dosage of 150 mg/m^2^ for patients enrolled in this study. The survival time (median PFS, 5.3 months; median OS, 10.3 months) in our cohort was similar to that of the Japanese phase II trial of FOLFIRINOX (median PFS, 5.6 months; median OS 10.7 months) as well as the FOLFIRINOX phase III trial (median PFS, 6.4 months; median OS, 11.1 months) [11].

In addition to considering more proper dosage, it would be important to investigate novel predictors for efficacy and survival outcomes of the FOLFOIRINOX treatment in pancreatic cancer. The predictive factor would enable physicians to distinguish patients who benefit more from the intensive chemotherapy with potentially severe toxicities. A subgroup analysis of the TRIBE trial evaluating a triplet regimen plus bevacizumab for colorectal cancer has reported ETS and DpR as promising surrogates for survival time [5]. The ETS, which is decrease in tumor load measured at the time of first imaging after the start of treatment, is considered to be an early indicator of sensitivity to chemotherapy for metastatic colorectal cancer. The DpR is defined as the percentage of tumor shrinkage, based on the longest diameters or reconstructed volume, observed at the lowest point, so called nadir, compared with baseline [4]. FOLFOXIRI plus bevacizumab improved the ETS and DpR when compared with FOLFIRI plus bevacizumab in a sub-analysis of the TRIBE trial [14]. Moreover, the analysis showed achieving rapid and deep tumor shrinkage consistently delayed tumor progression and prolonged survival in patients treated with the triplet-based regimen. Therefore, the ETS and DpR may become novel factors to predict favorable outcomes of FOLFIRINOX treatment in patients with advanced pancreatic cancer.

Our study could demonstrate for the first time that ETS to the intensive chemotherapy with triplet regimen may predict favorable outcomes in patients with advanced pancreatic cancer. The ETS was significantly associated with better PFS as well as OS in patients treated with the FOLFIRINOX therapy; moreover, the association had a statistically significance for PFS but a strong trend for OS in multivariate analysis. The responders had significantly longer OS compared to the non-responders in both univariate and multivariate analyses. In addition, the DpR was statistically significantly but weakly associated with OS but not PFS. The ETS is an assessment method that is usually judged at earlier timing from treatment start than response to chemotherapy and DpR; therefore, it may enable us to distinguish patients earlier, who benefit more from FOLFIRINOX therapy. Survival time is much shorter in pancreatic cancer compared to colorectal cancer, leading to shorter treatment duration. Thus, it may be reasonable to switch regimen for ETS-negative patients. Our findings and hypothesis need a prospective validation which may contain an adaptive trial design to evaluate treatment decision according to ETS status.

Our study was retrospective, and included a limited assessable population because of small number of patients. FOLFIRIXNOX regimen is usually used as a first-line treatment for advanced pancreatic cancer; therefore, the ETS and DpR should be investigated in patients receiving the triplet regimen as an initial therapy. We performed a sub-analysis according to treatment line; however, we failed to indicate the significant association between the biomarkers and clinical outcomes due to small sample size (*n* = 31) (data not shown). It is desirable to assess them prospectively for achieving more accurate results using larger patient cohorts. In previously reported trials which investigated association of the ETS with clinical outcomes, the tumor assessment was carried out every 8 weeks until the evidence of disease progression by means of computer tomography scan [4, 5, 15]; however, we measured target lesions for assessment of ETS at 6 or 8 weeks from treatment start due to retrospective trial and limited data, causing a difference in the timing of evaluation for ETS from previous reports. We therefore should integrate the term of evaluation for ETS in future studies.

In conclusion, the ETS and DpR were significantly associated with clinical outcomes in advanced pancreatic cancer patients treated with FOLFIRINOX chemotherapy. Our results also suggest that the early response to the triplet regimen may predict better clinical outcomes in patients with advanced pancreatic cancer. These findings are warranted to validate in future trials.

## MATERIALS AND METHODS

### Patient

This study enrolled advanced pancreatic cancer patients with an ECOG PS of 0 or 1, and adequate hematological, liver, and renal function, who received FOLFIRINOX as first- or second-line treatment between November 2012 and November 2015 in 3 institutions of Showa University (Showa University Northern Yokohama Hospital, Showa University Hospital, and Showa University Koto Toyosu Hospital). All clinical data were obtained retrospectively from their medical records.

### Treatment

We utilized the FOLFIRINOX with modified dosage according to the Japanese phase II trial [13]: irinotecan was also infused intravenous (i.v.) over 90 minutes at 150 mg/m^2^ and then 2 hours i.v. infusion of oxaliplatin at 85 mg/m^2^ and 2 hours i.v. infusion of l-leucovorin at 200 mg/m^2^ followed by continuous i.v. infusion of 5-FU over 46 hours at 2400 mg/m^2^. This regimen was repeated every two weeks until disease progression. Dose modifications were made at the treating physician's discretion. Patient received palonosetron, aprepitant, and dexamethasone for emesis prophylaxis.

### Study design

We retrospectively analyzed characteristics, RR, PFS, and OS in patients enrolled in this study. The objectives of our study were to evaluate the association of ETS with PFS and OS but also to address the correlation between clinical outcomes and DpR. Responses were evaluated according to the RECIST version 1.1 by the investigators. ETS was defined as a reduction ≥ 20% of target lesions' diameters measured at 6 to 8 weeks from treatment start. DpR was defined as the percentage of maximal tumor shrinkage observed at the nadir diameter compared with baseline. A DpR of negative 100% indicates the complete disappearance of all target tumor lesions. We thought it was difficult to take an accurate measurement of the primary site of tumor in patients with locally advanced pancreatic cancer. Then, we mainly evaluated metastatic measurable lesions in patients with metastatic pancreatic cancer for evaluation of ETS and DpR.

### Statistical analysis

Kaplan-Meier analysis was used to generate survival curves of PFS and OS. The median survival time was compared using the log-rank test. The Spearman's rank correlation coefficient was adopted to evaluate the association between DpR and survival including PFS and OS. Multivariate analysis was performed based on Wald test for PFS and OS in the multivariable Cox regression model adjusted for ECOG PS (0 vs. 1) and diagnosis (locally advanced vs. metastatic).

Statistical analyses were carried out using JMP 9.0.3 (SAS institute, Cary, NC, USA).
